# Room temperature synthesis of water-soluble spherical particles of a uniform diameter composed of carbon nanobelts and C_60_ molecules

**DOI:** 10.1038/s41598-022-19475-z

**Published:** 2022-09-08

**Authors:** Sieun Choi, Shunji Kurosu, Yuta Mashiko, Takanobu Minakawa, Toru Maekawa

**Affiliations:** 1 Graduate School of Interdisciplinary New Science, Toyo University, 2100, Kujirai, Kawagoe, Japan; 2 Bio-Nano Electronics Research Centre, Toyo University, 2100, Kujirai, Kawagoe, Japan

**Keywords:** Nanoscale materials, Nanoscale materials

## Abstract

A carbon nanobelt (CNB) is a loop of fused benzene rings and a C_60_ molecule is a football shaped fullerene composed of 60 carbon atoms. In this study, we synthesize uniform spherical particles composed of (6,6)CNBs and C_60_ molecules in 1,2-dichlorobenzene at room temperature via bottom-up self-assembly, setting the molar concentrations of (6,6)CNBs and C_60_ molecules at appropriate values, and find that the particles are monodisperse even in water. The present room temperature synthetic methodology may well be applied to the creation of nano/micro structures/materials using basic carbon nano units such as cycloparaphenylene (CPP, carbon nanorings) and fullerenes; e.g., C_60_, C_70_ and C_59_N.

## Introduction

A carbon nanobelt (CNB) is a loop of fused benzene rings and in a sense a cutout of a single-walled carbon nanotube. Various types of CNBs have been successfully synthesized in recent years^1–7^ and CNBs have been utilized for the development of practical devices^8–10^. A C_60_ molecule is a football shaped fullerene composed of 60 carbon atoms^11^. Patterns and structures formed by atoms, molecules and particles via bottom-up self-assembly are of great interest and importance not only from a scientific point of view but for the design and production of functional materials and devices, the size of which ranges from nano to macroscopic scales^12–14^. Colloidal particles are commonly used/utilized in mechanical, chemical and biophysical/biochemical/biomedical engineering, where size uniformity and mono dispersibility of the particles in the solvent, particularly in water, become crucial factors^15–23^. In this study, we investigate secondary structures formed by (6,6)CNBs^1,2^ and C_60_ molecules, which are dissolved in 1,2-dichlorobenzene (see Fig. S1 in the Supplementary Information for the molecular structure of a (6,6)CNB). We find that uniform spherical particles are formed by (6,6)CNBs and C_60_ molecules in 1,2-dichlorobenzene at room temperature via bottom-up self-assembly, setting the molar concentrations of (6,6)CNBs and C_60_ molecules at appropriate values, and furthermore those particles are monodisperse even in water. The present facile room temperature synthetic methodology may well be applied to the creation of nano/micro structures/materials using basic carbon nano units such as cycloparaphenylene (CPP, carbon nanorings) and fullerenes; e.g., C_60_, C_70_ and C_59_N^24^.

The solutions of (6,6)CNBs, C_60_ molecules and a mixture of (6,6)CNBs and C_60_ molecules dissolved in 1,2-dichlorobenzene are shown in Fig. S2 in the Supplementary Information. The colour of the solution of (6,6)CNBs dissolved in 1,2-dichlorobenzene was yellowish, whereas that of C_60_ molecules dissolved in 1,2-dichlorobenzene was deep purple as well known. The colour of the solution changed to brown after the solutions of (6,6)CNBs and C_60_ molecules had been mixed together.

**Figure 1:**
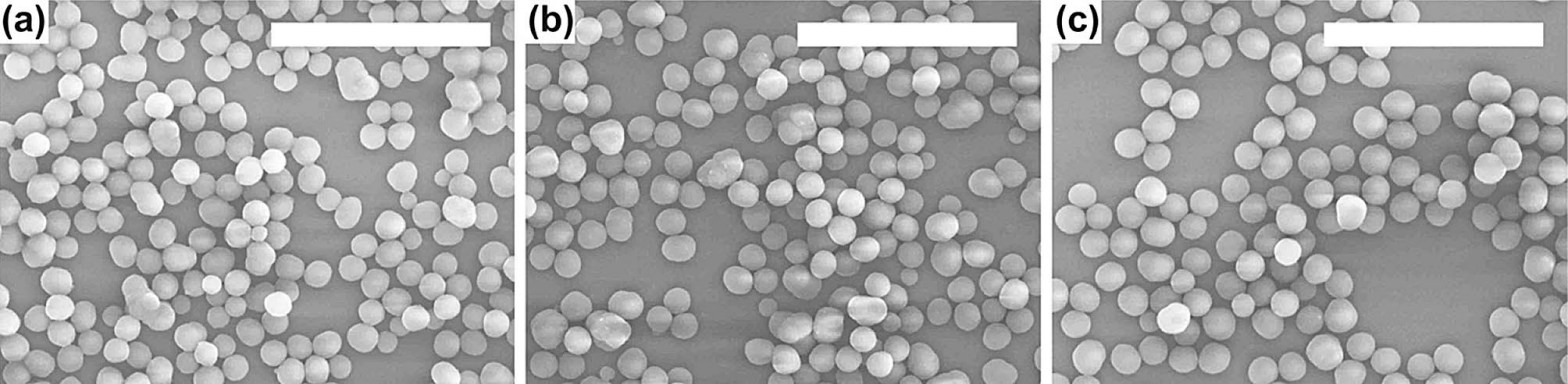
SEM images of particles formed by (6,6)CNBs and C_60_ molecules. The particles were synthesized setting the ratio of the molar concentration of (6,6)CNBs to that of C_60_ molecules dissolved in 1,2-dichlorobenzene at 1:2 (the concentrations of (6,6)CNBs and C_60_ molecules were 0.35 and 0.70 µmol ml^−1^). The scale bars represent 5 µm. (**a**) 3 h after the mixture of the two solutions; (**b**) 4 h; (**c**) 24 h.

**Figure 2:**
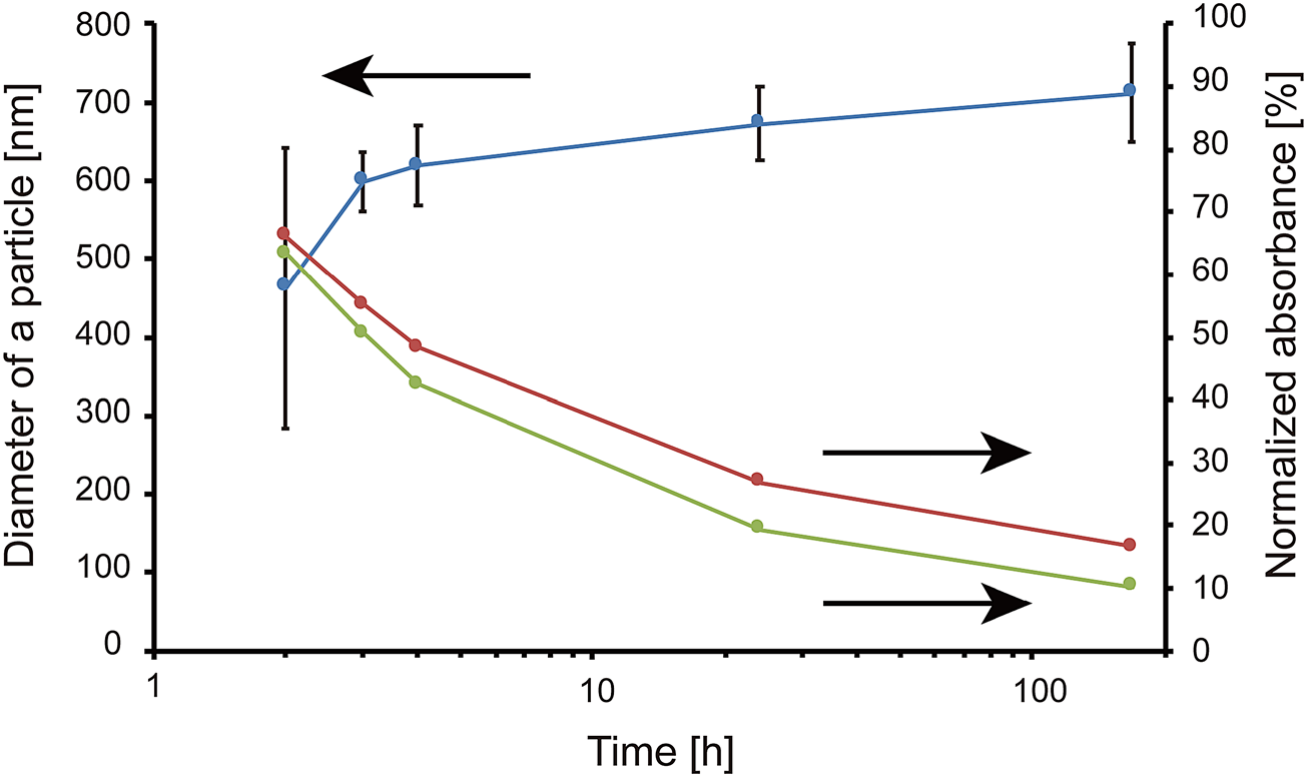
Time variation of the diameter of a particle and the peaks’ intensities of the absorption spectra corresponding to (6,6)CNBs and C_60_ molecules dissolved in 1,2-dichlorobenzene. The ratio of the molar concentration of (6,6)CNBs to that of C_60_ molecules is 1:2. Dotted red line—absorbance of 318 nm; dotted green line—absorbance of 329 nm; dotted blue line—average diameter of a particle.

We found that particles were produced in 1,2-dichlorobenzene after the mixture of the two solutions in all of the cases of different ratios of the molar concentration of (6,6)CNBs to that of C_60_ molecules (see Table 2 in the Methods for the actual concentrations of (6,6)CNBs and C_60_ molecules dissolved in 1,2-dichlorobenzene). However, smooth spherical particles of a uniform diameter were formed when the ratio of the molar concentration of (6,6)CNBs to that of C_60_ was set at 1:2 (the concentrations of (6,6)CNBs and C_60_ molecules were, respectively, 0.35 and 0.70 µmol ml^−1^). SEM images of particles formed by (6,6)CNBs and C_60_ molecules are shown in Fig. 1, where the ratio of the molar concentration of (6,6)CNBs to that of C_60_ was 1:2. The size of the particles increased with time (see also Fig. S3 in the Supplementary Information for the size distributions of the particles as a function of the time and Fig. 2 for the time variation of the diameter of a particle). Note that no particles were formed in the solution of (6,6)CNBs dissolved in 1,2-dichlorobenzene and in the solution of C_60_ molecules dissolved in 1,2-dichlorobenzene. The surface of the spherical particles was smooth and the diameter of a particle was uniform when the particles were synthesized setting the ratio of the molar concentration of (6,6)CNBs to that of C_60_ at 1:2 as mentioned, whereas the surface of the particles was uneven and the size of a particle varied when the ratio was different from 1:2 (see Fig. S4 in the supplementary Information for the size distributions and SEM images of particles produced when the ratio of the molar concentration of (6,6)CNBs to that of C_60_ was 1:1, 1:2 and 1:3).

The absorption spectra by the supernatant of the solution, in which a mixture of (6,6)CNBs and C_60_ molecules were dissolved in 1,2-dichlorobenzene, are shown in Fig. S5 in the Supplementary Information, where the ratio of the molar concentration of (6,6)CNBs to that of C_60_ molecules was changed (see Fig. S5a) in the Supplementary Information) and the time variation of the absorption spectra when the ratio of the molar concentration of (6,6)CNBs to that of C_60_ molecules was 1:2 is shown in Fig. S5b in the Supplementary Information. Note that the wavelengths of photons absorbed by (6,6)CNBs and C_60_ dissolved in 1,2-dichlorobenzene had been measured and those by (6,6)CNBs were 296, 318, 355, 373 and 396 nm, while those by C_60_ were 297, 329 and 407 nm (see Fig. S6 in the Supplementary Information for the absorption spectra by the individual solution of C_60_ and (6,6)CNBs dissolved in 1,2-dichlorobenzene). It is supposed that *m* × (6,6)CNBs and *n* × C_60_ molecules ((*m*, *n*) = (2, 1) and (1, 1 ~ 3) in the present study. See Methods Table 2) were combined to form a compound of ((6,6)CNB)_*m*_-(C_60_)_*n*_ in the solution since the absorption peaks corresponding to (6,6)CNBs and C_60_ decreased when the ratio of the molar concentration of (6,6)CNBs to that of C_60_ molecules was *m*:*n*, noting that smooth spherical particles of a uniform diameter were formed when the ratio of the molar concentration of (6,6)CNBs to that of C_60_ molecules was 1:2 as mentioned. The ratio of the molar concentration of (6,6)CNBs to that of C_60_ molecules was increased by increasing the molar concentration of C_60_ molecules, keeping the molar concentration of (6,6)CNBs constant (see Methods Table 2). When the ratio was 1:2, compounds composed of (6,6)CNB-(C_60_)_2_ were produced and spherical particles of a uniform diameter were formed by the compounds as mentioned above. However, there were still individual (6,6)CNBs, which did not participate in the formation of the compounds; (6,6)CNB-(C_60_)_2_, in the solution from a statistical point of view. As the ratio of the molar concentrations was increased to 1:2.25, 1:2.5, 1:2.75 and 1:3, it is supposed that those individual CNBs and C_60_ molecules formed different types of compounds such as (6,6)CNB-C_60_ in addition to (6,6)CNB-(C_60_)_2_, noting that the excess C_60_ molecules were also physically attached to those compounds, and as a result, the number of (6,6)CNBs decreased in the solution and therefore the absorption at 318 nm was reduced. Note that the shape of the particles was non-spherical and the size distribution was broad as shown in Fig. S4c in the Supplementary Information in the case of higher ratio of the molar concentrations. The intensity of the absorption spectra corresponding to (6,6)CNBs and C_60_ molecules decreased with time (see Fig. S5b in the Supplementary Information, where the ratio of the molar concentration of (6,6)CNBs to that of C_60_ molecules was set at 1:2). In other words, the number of compounds; (6,6)CNB-(C_60_)_2_, increased with time and those compounds formed spherical particles.

The time variation of the diameter of a particle and the peaks of the absorption spectra corresponding to (6,6)CNBs and C_60_ molecules in 1,2-dichlorobenzene are shown in Fig. 2, where the ratio of the molar concentration of (6,6)CNBs to that of C_60_ molecules was set at 1:2. The diameter of a particle increased with time, whereas the amount of (6,6)CNBs and C_60_ molecules in the solution decreased with time, which means that the number of (6,6)CNB-(C_60_)_2_ produced in the solution and the diameter of a particles formed by (6,6)CNB-(C_60_)_2_ increased with time as mentioned.

The mean diameter of a particle formed 168 h after the mixture of the two solutions and the hydrodynamic diameter and zeta potential of a particle dispersed in distilled water are shown in Table 1, where the particles were synthesized setting the ratio of the molar concentration of (6,6)CNBs to that of C_60_ molecules at 1:2. The diameter and hydrodynamic diameter of a particle synthesized 168 h after the mixture of the two solutions were quite uniform. Importantly, the absolute value of the zeta potential of a particle dispersed in water was so high as 38.8 mV that the particles were monodisperse even in water (see Fig. S7 and Video S1 in the Supplementary Information for the precipitation process of the solution and Fig. S8 in the Supplementary Information for the time variation of the turbidity of the suspension). Note that the particles eventually precipitated in water due to their own weight, but once the suspension had been shaken, the particles evenly dispersed again thanks to the high absolute value of the zeta potential in water (see Video S2 in the Supplementary Information).

**Table 1:** Diameter of a particle produced 168 h after the mixture of the two solutions, and the hydrodynamic diameter and zeta potential of a particle dispersed in distilled water.

Diameter^a^ [nm]	Hydrodynamic diameter^b^ [µm]	Zeta potential^c^ [mV]
(7.12 ± 0.63) × 10^2^	1.30 ± 0.09	− 38.8 ± 0.7

The particles were synthesized setting the ratio of the molar concentration of (6,6)CNBs to that of C_60_ molecules at 1:2 (the concentrations of (6,6)CNBs and C_60_ molecules were, respectively, 0.35 and 0.70 µmol ml^−1^).

^a^The diameter of the particles was measured, targeting at 100 particles from SEM images.

^b^The hydrodynamic diameter of a particle dispersed in distilled water was measured by Zetasizer.

^c^The zeta potential of a particle dispersed in distilled water was measured by Zetasizer.

The result of the thermogravimetric (TG) analysis of the particles is shown in Fig. S9 in the Supplementary Information. There was no significant weight loss and the overall weight loss was 0.47% (7 µg/1.485 mg). We suppose that 1,2-dichlorobenzene was not contained in the particles. Even if it had been contained in the particles, the amount of 1,2-dichlorobenzene would have been only 47 nmol in 1485 mg of particles. Note that the particles were also monodisperse in water even after the TG analysis, the zeta potential of the particles after the TG analysis being the same as that of the particles before the TG analysis.

The mass spectra of particles formed 168 h after the mixture of the two solutions are shown in Fig. S10 in the Supplementary Information, which indicates that (6,6)CNBs are positively charged, while C_60_ molecules are negatively charged. It is therefore supposed that the particles were formed by compounds composed of positively charged (6,6)CNBs and negatively charged C_60_.

We carried out some preliminary simulations concerning the structures formed by compounds [(6,6)CNB-(C_60_)_*m*_]_*n*_, where (*m*, *n*) = (1, 1), (2, 1), (2, 2) and (2, 3), based on a semi-empirical method; PM6^25^, according to which a compound; (6,6)CNB-(C_60_)_2_, can be stably formed, but triple compounds are not aligned in a regular form (see Fig. S11 in the Supplementary Information). Based on the experimental and numerical results, it is supposed that two C_60_ molecules and one (6,6)CNB were bonded via charge transfer to form compounds composed of C_60_—(6,6)CNBs—C_60_, noting that a peak around 204 nm in the absorption spectrum by the particles dispersed in ethanol was induced by charge transition (see Fig. S12 in the Supplementary Information).

A TEM image of a particle formed when the ratio of the molar concentration of (6,6)CNBs to that of C_60_ molecules was 1:2 is shown in Fig. S13 in the Supplementary Information. It is clearly shown that the particle is not formed by regularly oriented compounds. An XRD spectrum of the particles is shown in Fig. S14 in the Supplementary Information. There were no clear/sharp diffraction peaks and therefore it is supposed that the particles are formed by randomly oriented compounds composed of C_60_—(6,6)CNBs—C_60_.

In summary, particles composed of (6,6)carbon nanobelts and C_60_ molecules were synthesized via self-assembly at room temperature by mixing two solutions of (6,6)carbon nanobelts and C_60_ molecules dissolved in 1,2-dichlorobenzene. Smooth spherical particles of a uniform diameter were formed particularly when the ratio of the molar concentration of (6,6)CNBs to that of C_60_ molecules was set at 1:2 (the concentrations of (6,6)CNBs and C_60_ molecules were 0.35 and 0.70 µmol ml^−1^). It is supposed that compounds composed of C_60_—(6,6)CNBs—C_60_ were self-assembled in the solution, two C_60_ molecules and one (6,6)CNB having been bonded via charge transfer, and that the particles were formed by randomly oriented compounds. The absolute value of the zeta potential of the particles dispersed in distilled water was so high that the particles were monodisperse in water, which means that the particles may well be used as stable colloidal particles in water. The present synthetic methodology is so simple that it may also be applied to the creation of nano/micro structures/materials using basic carbon nano units such as [*n*]cycloparaphenylene (CPP, carbon nanorings) and fullerenes; e.g., C_60_, C_70_ and C_59_N.

## Methods

### Synthetic procedure of particles

We developed a facile room temperature methodology for producing particles composed of (6,6)CNBs and C_60_ molecules. The synthetic procedure is summarized below.

- (a)(6,6)CNBs (Tokyo Chemical Industry Co. Ltd.) and C_60_ molecules (Kanto Chemical Co. Inc.) were individually dissolved in 1,2-dichlorobenzene. The molar concentrations of (6,6)CNBs and C_60_ molecules dissolved in 1,2-dichlorobenzene are listed in Table 2.
- (b)Those two solutions were mixed, adding 2 ml of the solution of C_60_ molecules dissolved in 1,2-dichlorobenzene to 2 ml of the solution of (6,6)CNBs dissolved in 1,2-dichlorobenzene. The ratio of the molar concentration of (6,6)CNBs to that of C_60_ was set at 1:1, 1:1.25, 1:1.5, 1:1.75, 1:2, 1:2.25, 1:2.5, 1:2.75, 1:3 and 2:1 (see Table 2).
- (c)The mixed solutions were left still for 1, 2, 3, 4, 24 and 168 h at room temperature.
- (d)The solvent; i.e., 1,2-dichlorobenzene, was replaced by ethanol 1, 2, 3, 4, 24 and 168 h after the mixture of the two solutions, followed by sonication and centrifugation twice.
- (e)The particles separated by centrifugation were dispersed in distilled water, followed by sonication.

**Table 2:** Molar concentrations of the solution of CNBs and C_60_ dissolved in 1,2-dichlorobenzene.

Ratio of the molar concentrations of (6,6)CNBs and C_60_	Molar concentration of (6,6)CNBs [µmol ml^−1^]	Molar concentration of C_60_ [µmol ml^−1^]
1:1	0.70	0.70
1:1.25	0.70	0.875
1:1.5	0.70	1.05
1:1.75	0.70	1.225
1:2	0.70	1.40
1:2.25	0.70	1.575
1:2.5	0.70	1.75
1:2.75	0.70	1.925
1:3	0.70	2.10
2:1	1.40	0.70

The molar concentrations of (6,6)CNBs and C_60_ molecules became half after the mixture of the two solutions.

### Characterization and observation procedure

The following is the characterization and observation procedure.

- (a)The absorption spectra by the supernatant of the solution were measured by ultraviolet-visible (UV-Vis) spectroscopy (DU730, Beckman Coulter Inc.). The individual absorption spectrum of C_60_ and (6,6)CNBs dissolved in 1,2-dichlorobenzene was also measured in the same manner.
- (b)The structures of the particles were observed by scanning electron microscopy (SEM) (SU8030, Hitachi Ltd.) and transmission electron microscopy (TEM) (2200FS, JEOL Ltd.). The size of the particles was measured, targeting at 100 particles from the SEM images.
- (c)The hydrodynamic diameter and zeta potential of the particles dispersed in distilled water were measured by Zetasizer (Nano-ZS, Malvern Panalytical Ltd.).
- (d)The precipitation process of the particles dispersed in distilled water was observed, photographed and recorded on videotape. The intensity of the transmitted light of 500 and 600 nm wavelengths through the whole solution confined in a glass container was measured with a spectral photometer (U-3500 Spectrophotometer, Hitachi High-Tech Co.) and the turbidity, which was defined as $\left( {1 - I_{{trans}} /I_{{in}} } \right) \times 100\%$, where $I_{{in}}$ and $I_{{trans}}$ are, respectively, the intensities of the incident and transmitted light, was obtained.
- (e)Thermogravimetric (TG) analysis of the particles was carried out by a TG analyser (DTG-60, Shimadzu Corp.). The temperature was raised at a rate of 15.9 K min^−1^ up to 300 °C and the temperature was kept at 300 °C for 60 min with the flow of N_2_ gas. The measurement of the weight was calibrated with a precision scale (Excellence plus XP56, Mettler-Toledo International Inc.).
- (f)The molecular weight of the compounds forming the particles was measured with time-of-flight mass spectrometry (TOF-MS) (autoflex2, Bruker Co.).
- (g)The absorption spectrum by the particles dispersed in ethanol was measured by the UV-Vis spectroscopy (DU730, Beckman Coulter Inc.).
- (h)A droplet of the suspension of particles dispersed in ethanol was dropped onto a silicon-low background sample holder (M00016288, Rigaku Corp.) and X-ray diffractometric characterization of the particles was carried out by an X-ray diffractometer (SmartLab, Rigaku Corp.).

## Supplementary Information

Supplementary Information 1.

Supplementary Video 1.

Supplementary Video 2.

## Data Availability

All of the data supporting this work are available from the corresponding author upon reasonable request.
