# Supplementary material for: Room temperature synthesis of water-soluble spherical particles of a uniform diameter composed of carbon nanobelts and C60 molecules
Source: Sci Rep. 2022 Sep 8;12:15207. doi: 10.1038/s41598-022-19475-z (PMC9458716; doi:10.1038/s41598-022-19475-z)
Supplement: Supplementary file 1 — Supplementary Information 1. [file 41598_2022_19475_MOESM1_ESM.pdf]

Supplementary Information

Room temperature synthesis of water-soluble spherical particles of a uniform diameter composed of carbon nanobelts and C_{60} molecules

Sieun Choi[1,†], Shunji Kurosu[2,†], Yuta Mashiko[1,†], Takanobu Minakawa[1] and Toru Maekawa[1,2,*]

[1] Graduate School of Interdisciplinary New Science, Toyo University, 2100, Kujirai, Kawagoe, Japan

[2] Bio-Nano Electronics Research Centre, Toyo University, 2100, Kujirai, Kawagoe, Japan

[†] Equal contribution as the first author

* Corresponding author: maekawa@toyo.jp

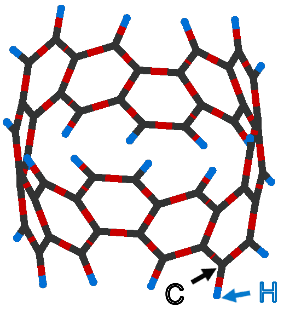

Figure S1 (6,6)carbon nanobelt.

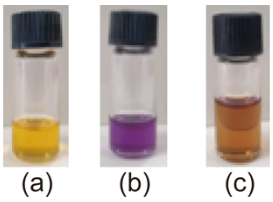

Figure S2 Solutions of (6,6)carbon nanobelts, C_{60} molecules and a mixture of (6,6)carbon nanobelts and C_{60} molecules dissolved in 1,2-dichlorobenzene. (a) (6,6)carbon nanobelts dissolved in 1,2-dichlorobenzene. The concentration of (6,6)carbon nanobelts is 0.70 μmol ml^{-1}. (b) C_{60} molecules dissolved in 1,2-dichlorobenzene. The concentration of C_{60} is 0.70 μmol ml^{-1}. (c) A mixture of (6,6)carbon nanobelts and C_{60} molecules dissolved in 1,2-dichlorobenzene. The concentrations of (6,6)carbon nanobelts and C_{60} are 0.35 μmol ml^{-1}.

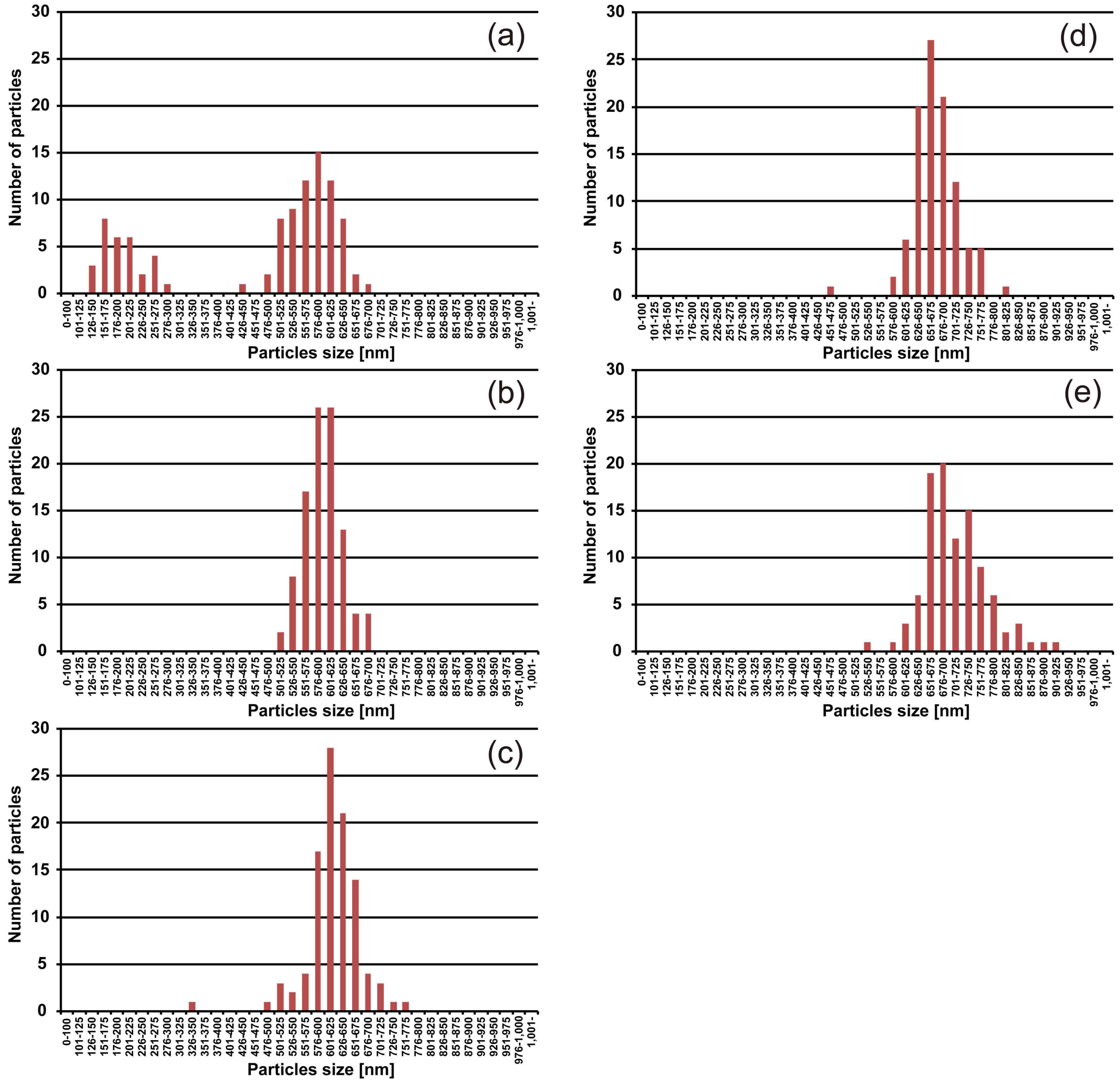

Figure S3 Size distributions of particles formed by (6,6)CNBs and C_{60} molecules. The concentrations of (6,6)CNBs and C_{60} molecules are, respectively, 0.35 and 0.70 μmol ml^{-1}. (a) 2 h after the mixture of the two solutions. The diameter of a particle is $(4.63 \pm 1.78) \times 10^2$ nm. (b) 3 h. $(5.99 \pm 0.38) \times 10^2$ nm. (c) 4 h. $(6.19 \pm 0.52) \times 10^2$ nm. (d) 24 h. $(6.73 \pm 0.47) \times 10^2$ nm. (e) 168 h. $(7.12 \pm 0.63) \times 10^2$ nm.

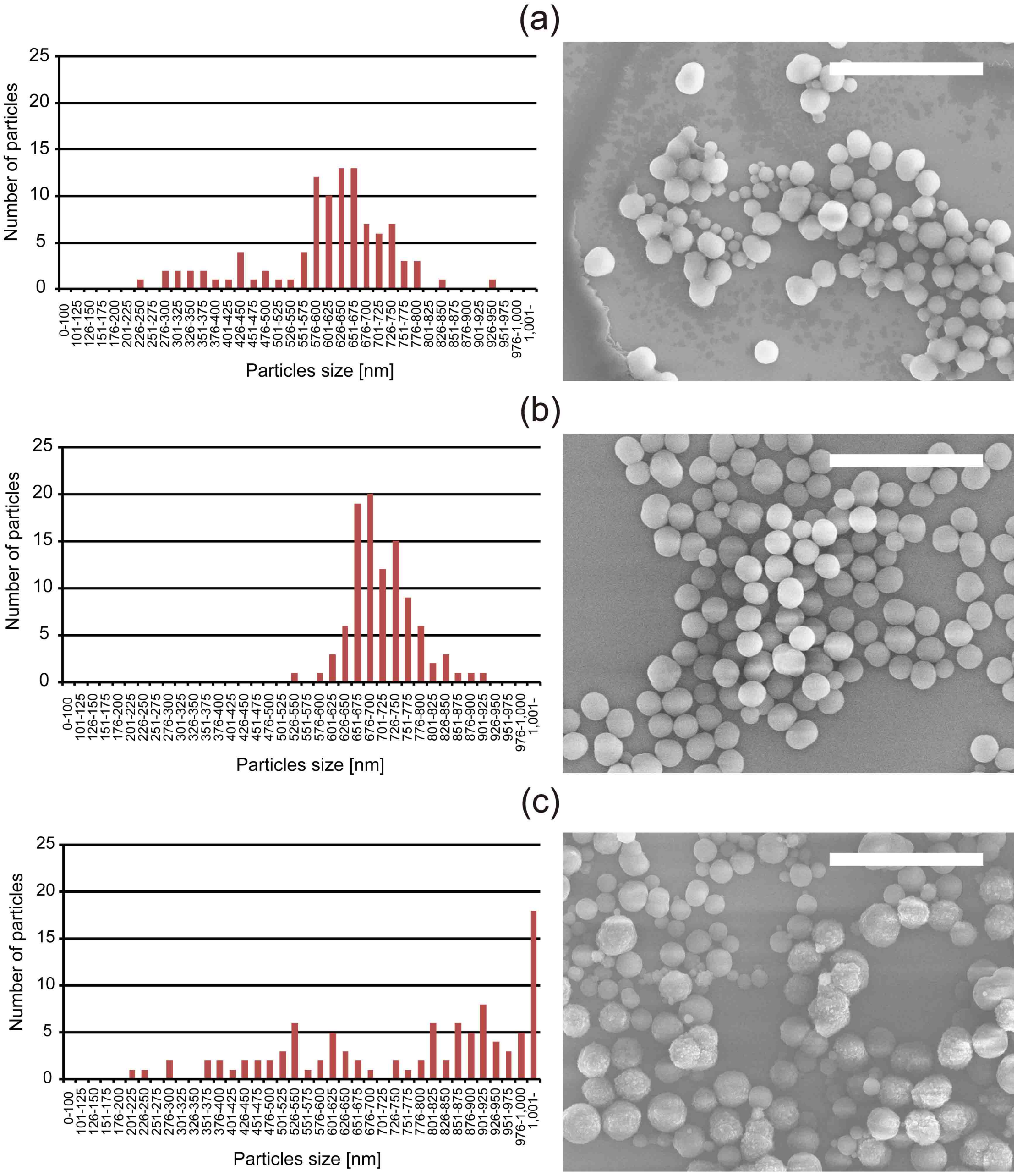

Figure S4 Size distributions sand SEM images of particles formed by (6,6)CNBs and C_{60} molecules 168 h after the mixture of the two solutions. (a) The ratio of the molar concentration of CNBs to that of C_{60} molecules in 1,2-dichlorobenzene is 1 : 1 (the concentrations of (6,6)CNBs and C_{60} molecules are 0.35 μmol ml^{-1}). The diameter of a particle is $(6.10 \pm 1.30) \times 10^2$ nm. (b) The ratio is 1 : 2 (the concentrations of (6,6)CNBs and C_{60} molecules are 0.35 and 0.70 μmol ml^{-1}). $(7.12 \pm 0.63) \times 10^2$ nm. (c) The ratio is 1 : 3 (the concentrations of (6,6)CNBs and C_{60} molecules are 0.35 and 1.05 μmol ml^{-1}). $(7.71 \pm 2.36) \times 10^2$ nm. The scale bars represent 5 μm.

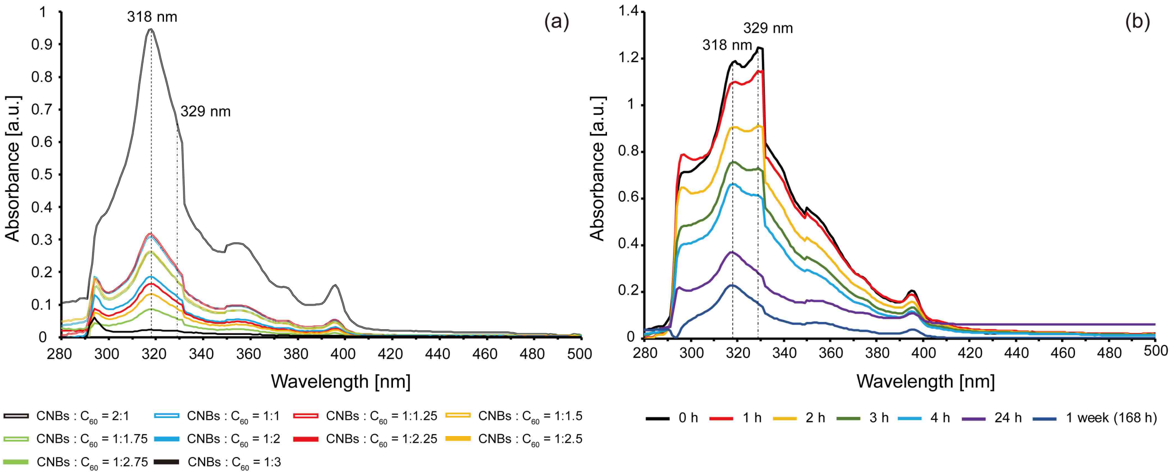

Figure S5 Absorption spectra by the supernatant of the solution. (6,6)CNBs and C_{60} molecules are dissolved in 1,2-dichlorobenzene. (a) Absorption spectra measured 168 h after the mixture of two solutions. The ratio of the molar concentration of CNBs to that of C_{60} is changed. (b) Time variation of the absorption spectra by the supernatant of the solution. The ratio of the molar concentration of CNBs to that of C_{60} is 1 : 2 (the concentrations of (6,6)CNBs and C_{60} molecules are 0.35 and 0.70 μmol ml^{-1}).

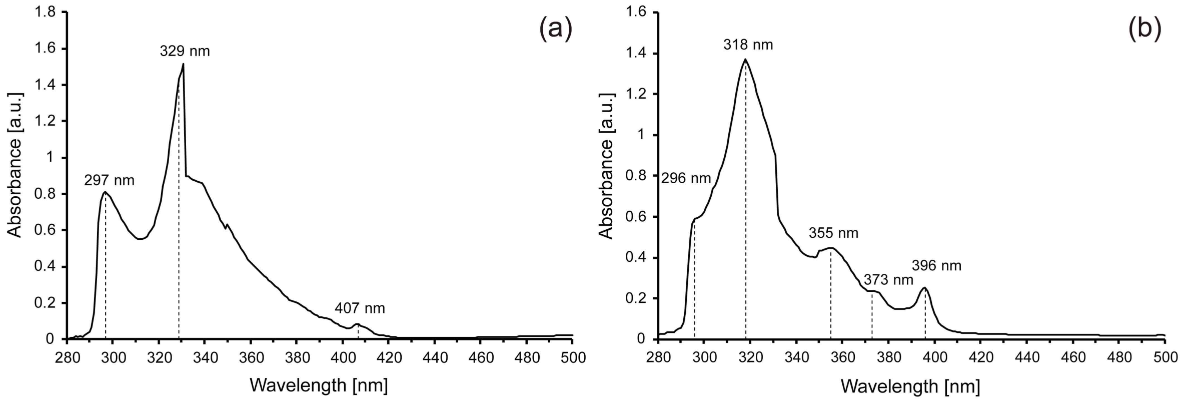

Figure S6 Absorption spectra by supernatant of the solution of (a) C_{60} molecules and (b) (6,6)CNBs dissolved in 1,2-dichlorobenzene.

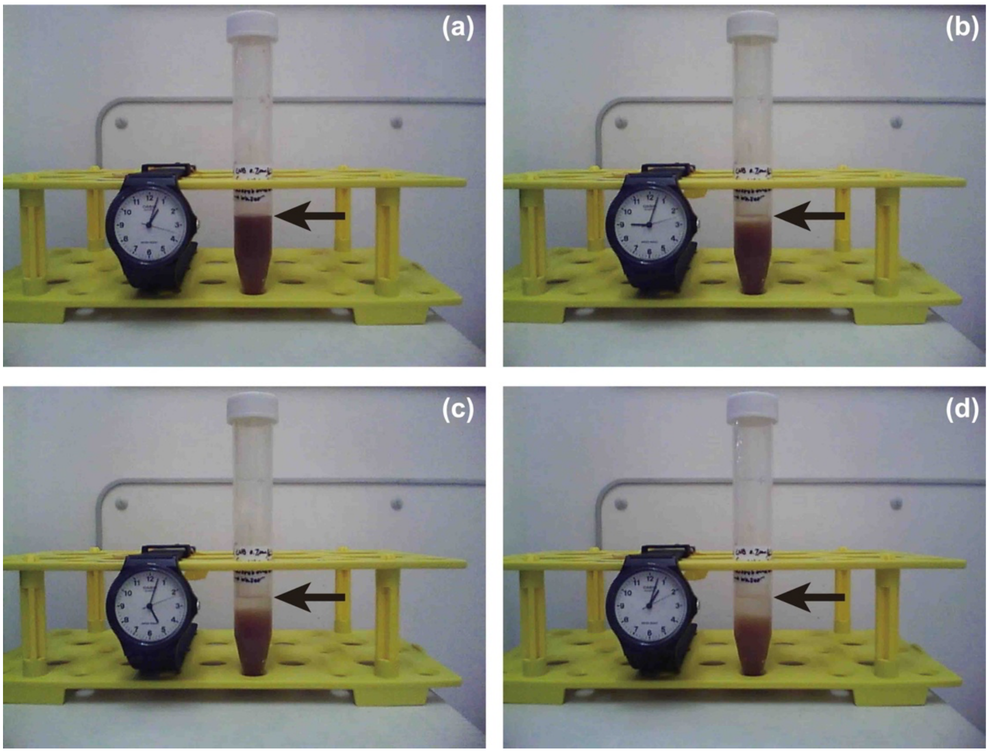

Figure S7 Snapshots of a suspension of particles formed by (6,6)CNBs and C_{60} molecules dispersed in distilled water. (a) The initial state; 0 h. (b) 8 h after dispersion. (c) 16 h. (d) 24 h. The arrows indicate the top of the solution.

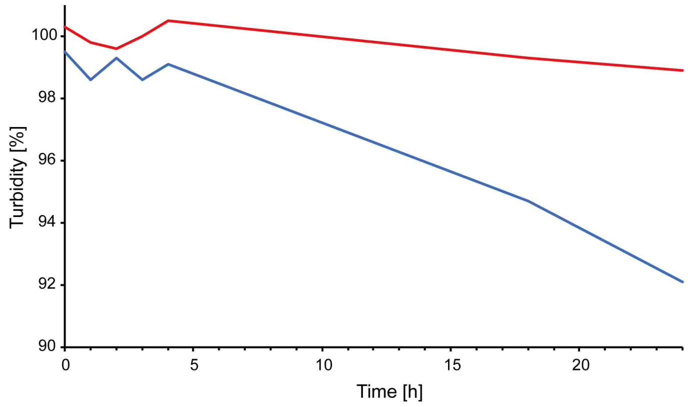

Figure S8 Time variation of the turbidity of the solution of particles dispersed in distilled water. The intensity of the transmitted light of 500 and 600 nm wavelength through the whole solution confined in a glass container was measured. The turbidity was defined as $(1 - I_{trans}/I_{in}) \times 100$ %, where I_{in} and I_{trans} are, respectively, the intensities of the incident and transmitted light.

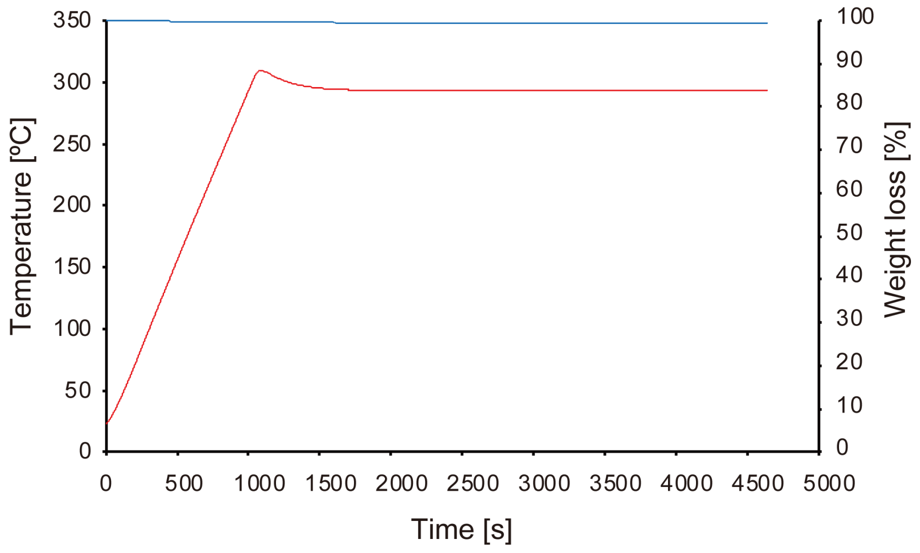

Figure S9 Time variation of the weight of samples and temperature. The temperature was raised at a rate of 15.9 K min^{-1} up to 300 °C and the temperature was kept at 300 °C for 60 min with the flow of N_2 gas. There was no significant weight loss, the overall weight loss having been 0.47 % (7 μg/1.485 mg).

Temperature [ºC] Weight loss [%]

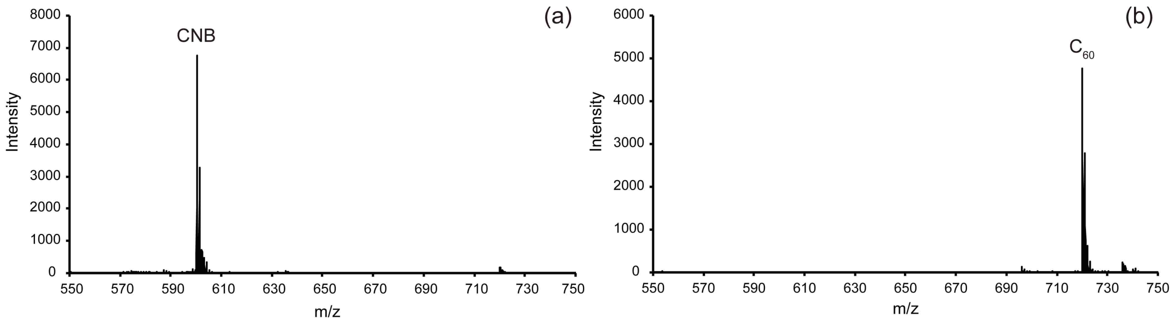

Figure S10 Mass spectra of the components forming particles. The particles formed 168 h after the mixture of the two solutions are targeted. (6,6)CNBs were positively charged, whereas C_{60} molecules were negatively charged. (a) (6,6)CNBs. (b) C_{60}.

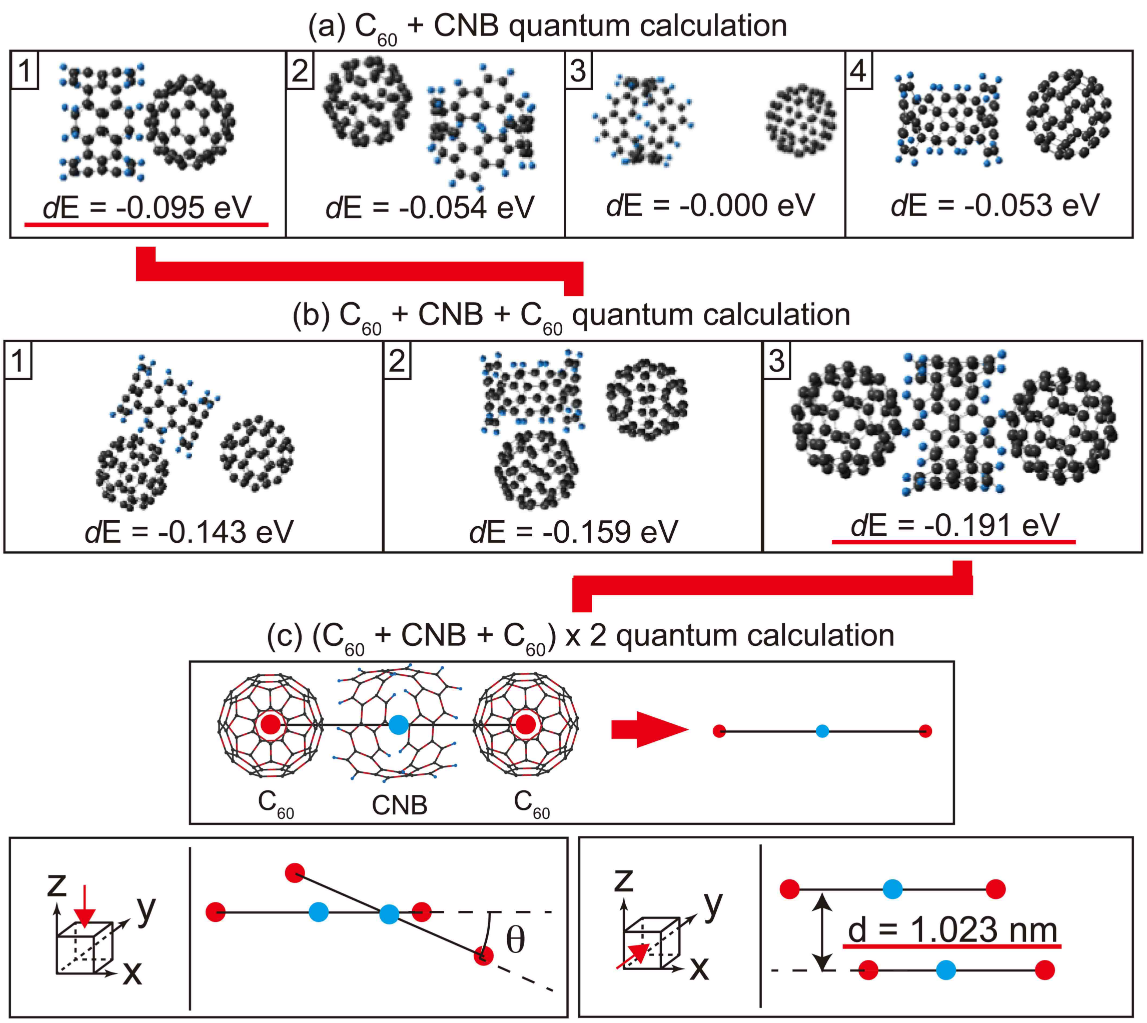

θ [°]	0	10	23.9822	30	40	50	60	70	80	90
*d*E [eV]	85.101	-0.499	-0.563	-0.559	-0.547	-0.534	-0.523	-0.514	-0.503	-0.464

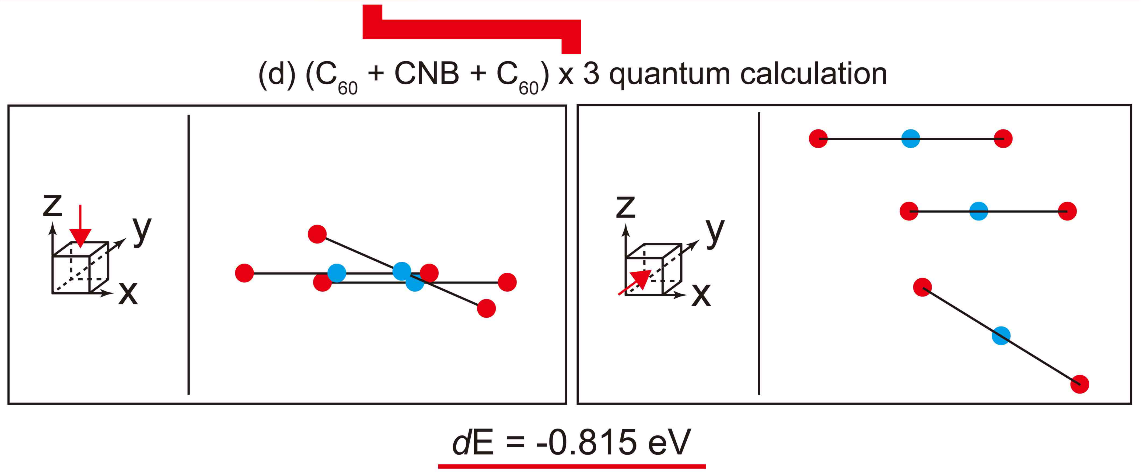

Figure S11 Compounds composed of (6,6)CNB and C_{60} molecules calculated by the PM6 method. (a) Compound formed by one (6,6)CNB and one C_{60} molecules; (6,6)CNB-C_{60}. (b) Compound formed by one (6,6)CNB and two C_{60} molecules; (6,6)CNB-$(C_{60})_2$. (c) Configuration of $[(6,6)CNB\text{-}(C_{60})_2]_2$. (d) Configuration of $[(6,6)CNB\text{-}(C_{60})_2]_3$.

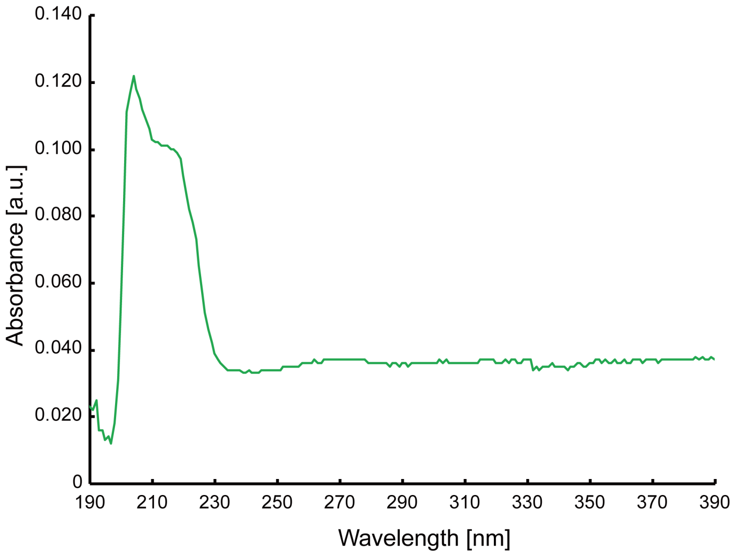

Figure S12 Absorption spectrum by particles dispersed in ethanol. It is supposed that the absorption peak around 204 nm is induced by charge transition in the compounds composed of C_{60} - (6,6)CNBs - C_{60}.

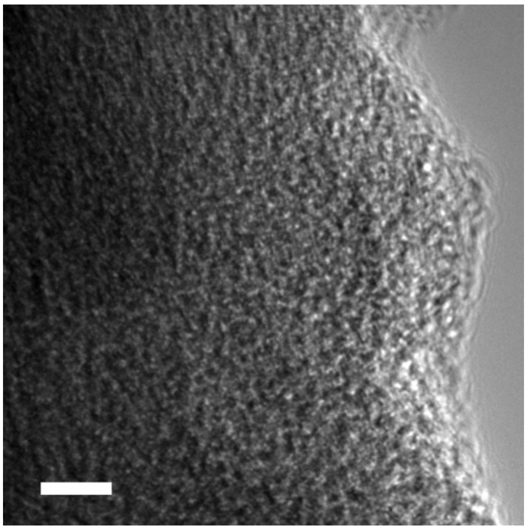

Figure S13 TEM image of a particle composed of (6,6)CNBs and C_{60} molecules. The particle was synthesized setting the molar concentration of (6,6)CNBs and C_{60} molecules, respectively, at 0.35 and 0.7 μmol ml^{-1}. The scale bar represents 5 nm.

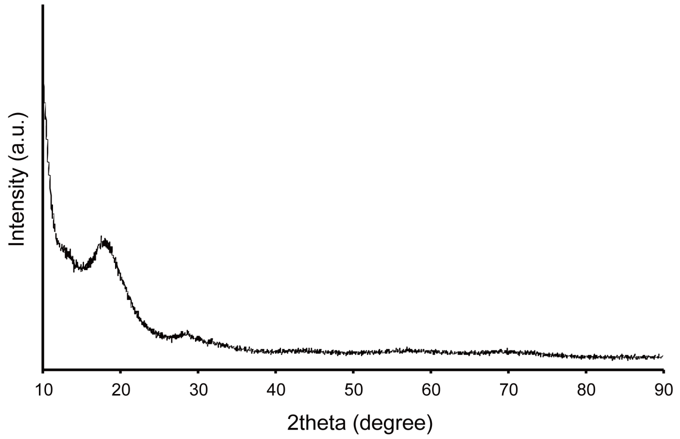

Figure S14 XRD spectrum of particles formed by compounds composed of (6,6)CNBs and C_{60} molecules. The particles were synthesized setting the molar concentration of (6,6)CNBs and C_{60} molecules, respectively, at 0.35 and 0.7 μmol ml^{-1}.

Video-1.mp4 Time variation of the precipitation process of the particles dispersed in distilled water from 0 up to 24 h. The arrow indicates the top of the suspension. Although the particles gradually precipitate due to their weight, they can be monodispersed again once the suspension is shaken (see Video-2.mp4).

Video-2.mp4 Monodispersibility of the particles in distilled water. The particles were forced to precipitate on the bottom of the microtube by centrifugation. The particles were monodispersed again in distilled water without any agglomeration by shaking the suspension by hand thanks to the high absolute value of the zeta potential of the particles.
